# Ferromagnetism modulation by ultralow current in a two-dimensional polycrystalline molybdenum disulphide atomic layered structure

**DOI:** 10.1038/s41598-022-22113-3

**Published:** 2022-10-13

**Authors:** Iriya Muneta, Takanori Shirokura, Pham Nam Hai, Kuniyuki Kakushima, Kazuo Tsutsui, Hitoshi Wakabayashi

**Affiliations:** 1 Department of Electrical and Electronic Engineering, Tokyo Institute of Technology, Yokohama, Japan; 2 Department of Electrical and Electronic Engineering, Tokyo Institute of Technology, Tokyo, Japan; 3 Laboratory for Future Interdisciplinary Research of Science and Technology, Tokyo Institute of Technology, Yokohama, Japan

**Keywords:** Magnetic properties and materials, Two-dimensional materials, Electronic devices

## Abstract

Layered materials, such as graphene and transition metal dichalcogenides, are able to obtain new properties and functions through the modification of their crystal arrangements. In particular, ferromagnetism in polycrystalline MoS_2_ is of great interest because the corresponding nonmagnetic single crystals exhibit spontaneous spin splitting only through the formation of grain boundaries. However, no one has reported direct evidence of this unique phenomenon thus far. Herein, we demonstrate ferromagnetism modulation by an ultralow current density < 10^3^ A/cm^2^ in 7.5-nm-thick polycrystalline MoS_2_, in which magnetoresistance shows three patterns according to the current intensity: wide dip, nondip and narrow dip structures. Since magnetoresistance occurs because of the interaction between the current of 4*d* electrons in the bulk and localized 4*d* spins in grain boundaries, this result provides evidence of the current modulation of ferromagnetism induced by grain boundaries. Our findings pave the way for the investigation of a novel method of magnetization switching with low power consumption for magnetic random access memories.

## Introduction

To develop spintronic devices and integrated circuits, such as magnetic random access memories, it is necessary to use current to generate an effective magnetic field for the manipulation of the individual magnetization in each element. To date, many investigations have given us clear evidence showing that electrical current can modulate the magnetization direction in magnetic tunnel junctions through spin transfer torque^1,2^, spin–orbit torque^3^, and the spin Hall effect in heavy 5*d* metal wires^4,5^, which realizes magnetic random access memories with low energy consumption. However, magnetization manipulation still requires a large current density (> 10^5^ A/cm^2^). This is probably because typical ferromagnetic materials consist of 3*d* transition metals, in which intra- and interatomic exchange interactions of spins are too strong to interplay with electrical current.

The rearrangement of two-dimensional (2D) layered crystals is very important because novel properties and functions emerge that the bulk form of the corresponding material does not have. For example, bilayer graphene shows superconductivity^6^ and Hofstadter’s butterfly^7^ by twisting the orientation of the upper layer; a 2D single-layer ferromagnet changes into an antiferromagnet by stacking one more layer^8^; and transition metal dichalcogenide MoS_2_ shows memristive behaviour by connecting misoriented sheets that form grain boundaries (GBs)^9^. Moreover, MoS_2_ is a not magnetic material in pure single-crystal form, but it has been found to show ferromagnetism in polycrystalline structures, edge-rich nanowires, and defective structures^10–19^. Additionally, theoretical analysis indicates that imperfections in MoS_2_ crystals, such as point defects and topological defects, induce spin polarization in the 4*d* electrons of Mo atoms^20,21^. Since both conduction electrons and localized spins consist of mainly Mo 4*d* electrons, they would easily interact with each other because of the same orbital and relatively weaker intra- and interatomic exchange interactions in 4*d* spins compared to those of 3*d* ferromagnets. Thus, it can be estimated that magnetization will be easily modulated using electrical current in polycrystalline ferromagnetic MoS_2_. In this study, we demonstrate the current-induced modulation of ferromagnetism in a polycrystalline MoS_2_ film with several nanometre grains^22,23^ deposited by sputtering method, which produces a high density of GBs inducing ferromagnetism that we previously reported^11^. We measure the magnetic field *H* dependence of the magnetoresistance (MR) by applying a widely ranging current (1 nA–0.562 mA), by which we find that the MR curves show a variety of changes depending on the current intensity. Positive MR is mainly observed because of the spin-split band structure, and thus, we can estimate the magnetization properties, such as the coercive force, the magnetic field for magnetization saturation and the spin-split band structure inducing ferromagnetism. Additionally, linear MR is observed, which depends on the direction of the applied magnetic field.

**Figure 1:**
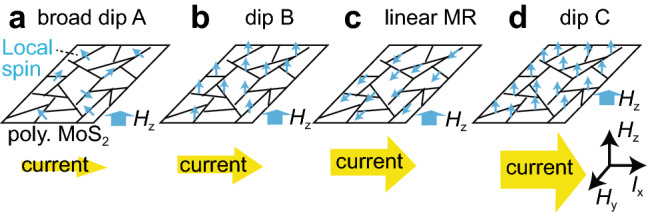
Schematic of the localized spin states in our magnetoresistance measurements. (**a**) In the small current applied, broad MR curves with dips A & A′ are observed in the *H*_z_ measurement because of the low spin density with disorder. (**b**) As the current increases, dip B with a large MR ratio is observed because of the high spin density along the *H*_z_ direction. (**c**) With a greater increase in current, the positive MR disappears, and flat or linear MR is observed because of the current-induced spin–orbit effective magnetic field along the *H*_y_ direction. (**d**) With a further increase in current, dip C is observed because of the high spin density, in which magnetization is along the *H*_z_ direction.

Figure 1 shows a schematic representing the localized spin states in the *H*_z_ (out of plane) measurement. When the current is small, the spin density is not high enough to form magnetization in the whole film, so a magnetic field is needed to align the localized spins (Fig. 1a). As the current increases, current electrons start to be trapped at the GBs, which leads to an increase in the spin density and results in the spontaneous formation of film magnetization (Fig. 1b). As the current increases further, a current-induced magnetic field is effectively applied to the localized spins because of the spin-dependent scattering at the GBs, which results in the alignment of the localized spins along the *H*_y_ (in-plane) direction (Fig. 1c). With a further increase in current, the magnetization aligns along the *H*_z_ direction again because of a further increase in the localized spins in the GBs (Fig. 1d). The current density is estimated to be approximately 7 × 10^–3^ A/cm^2^–4 × 10^3^ A/cm^2^ by dividing 1 nA–0.562 mA by the area defined by a 1.8 mm width and 7.5 nm depth. Considering the critical current of magnetization switching in magnetic tunnel junctions (> 10^5^ A/cm^2^)^2,24–28^, our MR modulation is successfully achieved at ultralow current densities (< 10^3^ A/cm^2^), even though the spin–orbit interaction in 4*d* electrons is weaker than that in 5*d* electrons, which provides evidence that current electrons easily interact with the localized 4*d* spins in the GBs in a polycrystalline MoS_2_ film. Our results place focus on the future investigation of 4*d* ferromagnets^29^, polycrystalline ferromagnetic semiconductors, and recently discovered 2D ferromagnets^8,30–34^, by controlling and manipulating ferromagnetism via basic electronics with low power consumption^35–39^.

## Results and discussion

In our previous study, out-of-plane 2*θ*–*θ* X-ray diffraction was measured^40^, in which MoS_2_ (002) peak was observed at 2*θ*  = 13.5°. Using Bragg’s law, we can estimate the layer distance of MoS_2_ *c* = *n* × λ/2sin*θ *= 6.56 Å. Here, we use *n* = 1 and λ = 1.5418 Å (wavelength of Cu*K*α). It is confirmed that this value is near the layer distance of bulk MoS_2_ (*c* = 6.15 Å)^41^. The observed MoS_2_ (002) peak is broad because our sample is polycrystal with imperfection of periodicity and incomplete flatness of van deer Waals surface.

Also, in our previous study, height distribution of MoS_2_ surface fabricated by sputter in the similar condition was measured using atomic force microscopy, in which root mean square (RMS) was 0.515 nm^42^. Compared to monolayer MoS_2_ by the chemical vaper deposition, this value is not bad because it is less than two layers distance 0.656 nm. Thus, our polycrystal MoS_2_ keeps van der Waals horizontal structure.

In addition, cross sectional lattice structure in polycrystal MoS_2_ was observed using the transmission electron microscopy (TEM) in our previous study^11^, from which we guess that the grain size is 5 nm. Based on this value, we can estimate the ratio of Mo atoms along GBs as follows. We assume *x* cm × *y* cm polycrystal monolayer MoS_2_, in which grains are regular squares with 5 nm edge length and are arranged like the grid of a checkerboard. The length of GBs is *x* cm/5 nm × *y* cm × 2 = 4*xy* × 10^6^ cm, while the Mo density in GBs is 1/3.16 Å = 1/3.16 × 10^8^ cm^−1^, in which we assume that Mo-Mo distance in GBs is 3.16 Å. Thus, the number of Mo atoms along GBs is 1/3.16 × 10^8^ cm^-1^ × 4*xy* × 10^6^ cm = *xy* × 1.27 × 10^14^ atoms. In single crystal monolayer MoS_2_ with *a* = 3.16 Å^41^, the Mo density is 1.16 × 10^15^ cm^−2^, and thus, the number of Mo atoms in the bulk area of *xy* cm^2^ is *xy* × 1.16 × 10^15^ atoms. Therefore, the ratio of Mo atoms along GBs and in bulk is (*xy* × 1.27 × 10^14^)/(*xy* × 1.16 × 10^15^) = 11%. This result agrees with the density of magnetic Mo atoms 0.61–16% estimated from the saturation magnetization 1–26 emu/cm^3^ reported in our previous study (see “Calculation for the density of magnetic Mo atoms” in “Methods” section)^11^.

Moreover, TEM image in Ref.^11^ provides us information about surface morphology: The most surface layer is incontiguous and interrupts by several nm. This surface morphology agrees with RMS value 0.515 nm from the AFM measurement^42^. See Supplementary Fig. 1 for the Raman measurement, the temperature dependence of resistance and the voltage-current characteristic.

**Figure 2:**
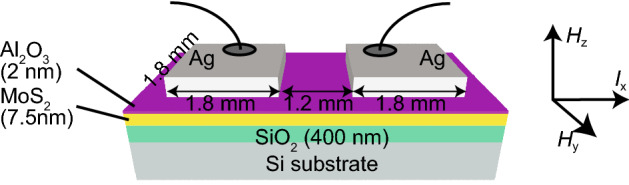
Schematic sample structure examined in our magnetoresistance measurement. Current is applied along the *I*_x_ direction between the two Ag contacts formed at the surface. The in-plane (out-of-plane) magnetic field is applied along the *H*_y_ (*H*_z_) direction.

**Figure 3:**
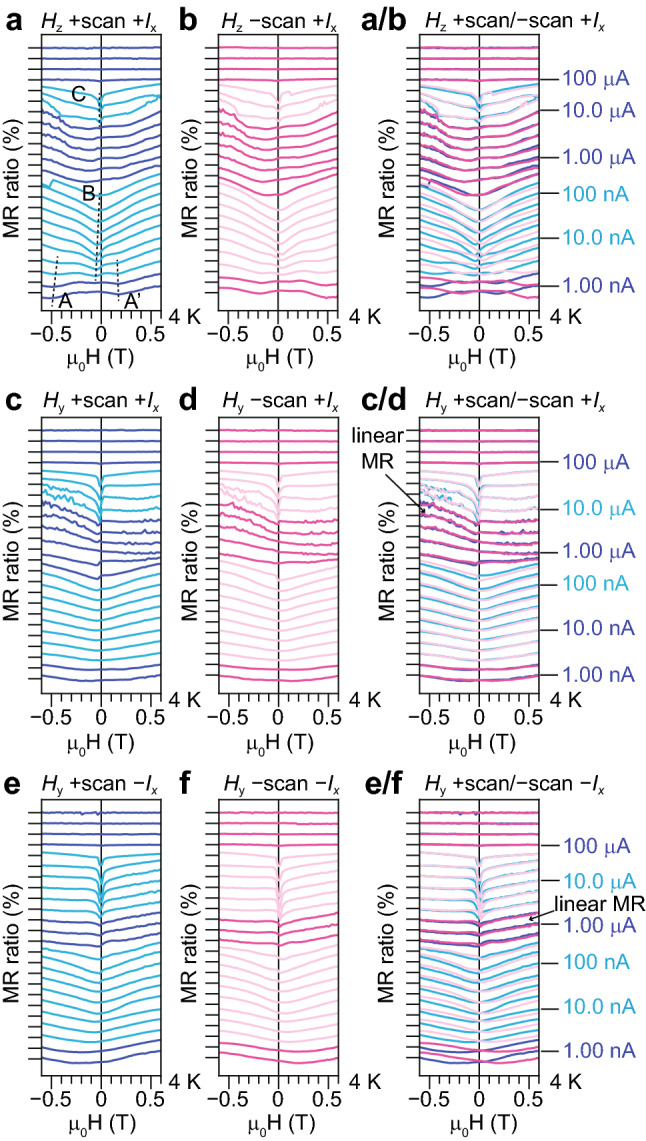
Magnetoresistance measured with various current intensities at 4 K. The current increases in steps of 1.78 times. (**a**,**b**) Magnetic field is applied along the out-of-plane direction (*H*_z_). (**c**–**f**) Magnetic field is applied along the in-plane direction crossing the current (*H*_y_). The current directions are + *I*_x_ (**a**–**d**) and − *I*_x_ (**e**,**f**). The magnetic field is swept from positive to negative (+ scan) in (**a**,**c**,**e**) and from negative to positive (− scan) in (**b**,**d**,**f**). (**a**/**b**,**c**/**d**,**e**/**f**) are superimposed together with + scan and − scan curves. The tick on the left axis indicates 0 in each curve. The tick space indicates 2%.

**Figure 4:**
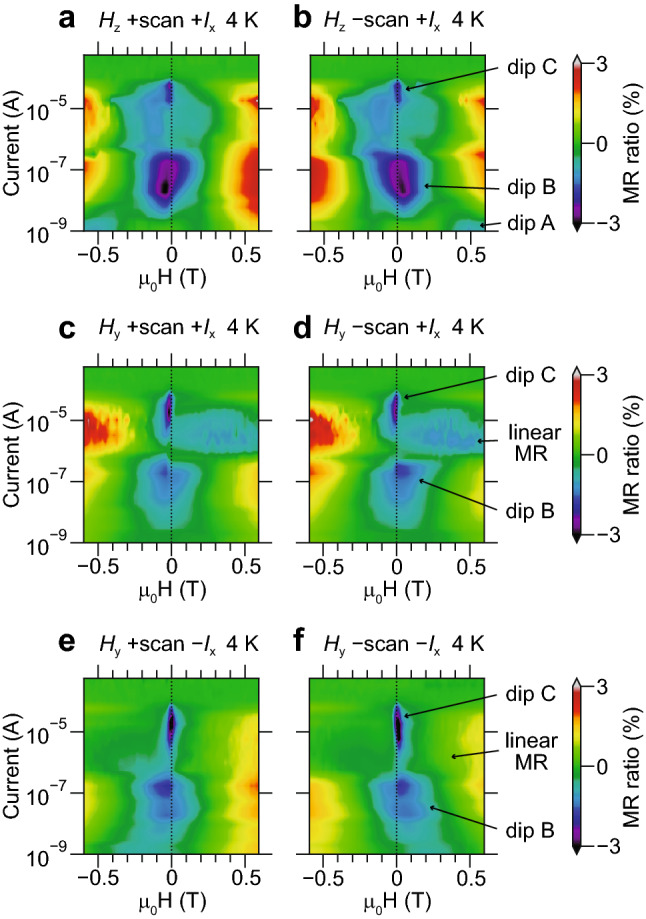
Colour-coded map of magnetoresistance measured with various currents at 4 K. (**a**,**b**) Magnetoresistance as functions of current and magnetic field applied along the out-of-plane direction (*H*_z_). (**c–f**) Magnetoresistance as functions of current and magnetic field applied along the in-plane direction crossing the current (*H*_y_). The current directions are + *I*_x_ (**a**–**d**) and − *I*_x_ (**e**,**f**). The magnetic field is swept from positive to negative (+ scan) (**a**,**c**,**d**) and from negative to positive (− scan) (**b**,**d**,**f**).

We analyse the details of the MR data measured in the two terminal device shown in Fig. 2. The MR-*H* curves measured at various currents are shown as multiple-curve plots in Fig. 3, where these curves are summarized according to the directions of *H* and the current. These curves are transformed into the colour-coded maps shown in Fig. 4. The MR-*H* curves mainly show positive MR curves, and a linear MR feature is observed in the *H*_y_ measurement. The positive MR curves are divided into three types: dips A and A′ with a large offset of *H*, dip B with a wide shape and dip C with a sharp shape. These observations indicate that the ferromagnetism in polycrystalline MoS_2_ is modulated by the applied current.

**Figure 5:**
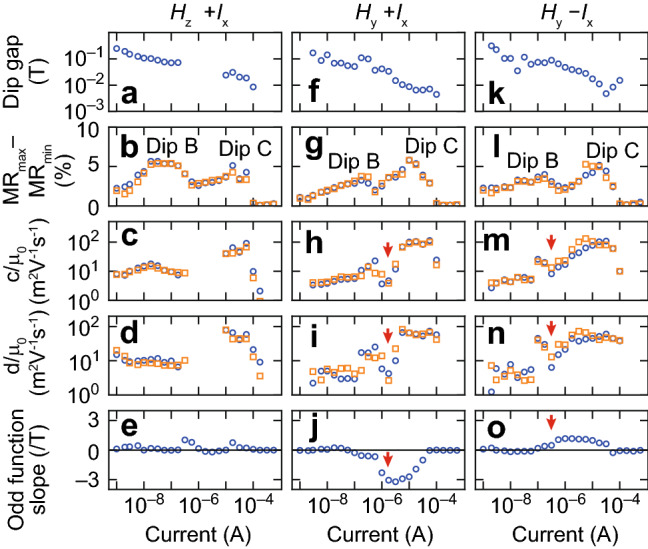
Dip gap, MR ratio, c value, d value and odd function slope obtained from our magnetoresistance measurements. (**a**–**e**) Data obtained in *H*_*z*_ + *I*_x_ measurement. (**f**–**j**) Data obtained in *H*_y_ + *I*_x_ measurement. (**k**–**o**) Data obtained in *H*_*y*_ − *I*_*x*_ measurement. Blue circles and orange squares in (**b**–**d**,**g**–**i,l**–**o**) correspond to + scan and − scan, respectively.

From the measurement data, we extract the physical values: the dip gap between + scan and -scan for dips B and C, MR_max_ − MR_min_, the c and d values of dips B and C obtained by fitting to the Khosla–Fischer equation^43^, and the slope of the odd function as a function of current, as shown in Fig. 5 (see “Methods” section for detail). The Khosla–Fischer equation represents positive MR based on up- and down-spin band model^43^, in which the parameters c and d are described by mobility and conductivity of up- and down-spin bands^44–47^. According to Takiguchi et al., conductivity can be described by the band energy, and thus, the spin split energy can be obtained from the d value by using this semi-empirical model (see “Calculation for spin split energy” in “Methods” section)^48^. Also, magnetic field for the saturation magnetization *H*_s_ can be evaluated numerically, so we can analyze it as a function of the current intensity.

As shown in Fig. 5a,f,k, the dip gap between + scan and − scan, which corresponds to coercive force *H*_c_, decreases 1/30 times as the current increases. A similar behaviour is seen in Ga_1−*x*_Mn_*x*_As, where the coercive force significantly decreases as the magnetic impurity *x* increases^49^. Based on this analogy, we can suppose that the density of spin magnetic moments increases as the current increases in polycrystalline MoS_2_.

As shown in Fig. 5d,i,n, the d value, which corresponds to the dip width and correlates to *H*_s_, is 20 times larger for dip C than for dip B. This behaviour is also seen in the Ga_1−*x*_Mn_*x*_As case reported in Ref.^49^, where *H*_s_ decreases as *x* increases. The dip gap and d value data indicate that the ferromagnetic properties are changed only by increasing the current without changing the magnetic doping required in typical ferromagnetic semiconductors.

The magnetic properties *H*_c_ and *H*_s_ are influenced by the density of magnetic atoms or distance between neighboring spins. Thus, similar density is one of the reasons of comparable behaviour of *H*_c_ and *H*_s_ in these two materials. The density of magnetic atoms of GaMnAs in Ref.^49^ is 0.5% and 7.4%. Meanwhile, the density of the magnetic atoms in our polycrystal MoS_2_ is estimated to be 0.61–16% using saturation magnetization 1–26 emu/cm^3^ measured on polycrystal MoS_2_ fabricated in the similar condition in our previous study (see “Calculation for the density of magnetic Mo atoms” in “Methods” section)^11^. Additionally, the similar mechanism of ferromagnetism in these two materials supports the comparison of the descriptions of *H*_c_ and *H*_s_. The mechanism of ferromagnetism in GaMnAs is carrier mediated exchange interaction between 3*d* localized spins, while the presumed mechanism of ferromagnetism in polycrystal MoS_2_ is carrier assisted exchange interaction between 4*d* localized spins in GBs. It is noted that *H*_c_ decreases has been observed in InMnAs as the increase in MnAs fraction^50^.

Additionally, we can estimate the spin-split energy of the 4*d* band from the d values^44,48^, which are 2.5 meV and 100 meV for dips B and C, respectively (see “Calculation for spin split energy” in “Methods” section for details). Although this estimation includes many assumptions, the derived values are worth considering for the band structure of polycrystalline MoS_2_ because the order of these values is near that of the theoretical calculation^20,21^.

The ferromagnetism enhancement by current probably occurs because the charges are trapped at the spin-dependent 4*d* sites in the Mo atoms in the GBs or because the itinerant carrier density increases in the long channel.

Linear MR starts to be observed at 3.9 A/cm^2^ (0.56 μA) (see in Fig. 3c/d,e/f to check which curves are linear MR). If the efficiency of the equivalent field because of the spin–orbit torque is the same as GaMnAs [99 Oe/(1 MA/cm^2^)]^3^, the effective magnetic field at 3.9 A/cm^2^ is 0.39 mOe. This value is quite small to change magnetic behaviour in the sample, and thus, the mechanism of MR change is thought to be different from the spin–orbit torque observed in 3*d* metal ferromagnets and ferromagnetic semiconductors. Conceivable mechanism of linear MR is the change of occupancy in 4*d* orbitals of Mo atoms in GBs because of spin-dependent scattering and the change of electron density in the long channel. The change of occupancy results in the change of the exchange interaction between localized 4*d* spins and results in the change of magnetic anisotropy because of spin–orbit interaction between spins and 4*d* orbitals with the magnetic quantum number.

In Ref.^51^, linear MR was observed in SmCo_5_ when the magnetization was independent of the magnetic field. This is described by Equation ***j*** = A(***M***·***H***)***E*** with steady ***M*** when ***H*** is swept, where ***j***, A, ***M*** and ***E*** are the current vector, coefficient, magnetization vector and electric field vector, respectively. As far as this theory is concerned, our observation of linear MR in the *H*_*y*_ measurement indicates that ***M*** is fixed to in-plane directions because of the spin–orbit effective magnetic field and the change of the exchange interaction induced by the current (Fig. 1c). This hypothesis is confirmed by the three pieces of experimental evidence below. One: When flipping the current direction, the odd function shows the opposite sign of slope (see Fig. 5j,o and “Calculation for linear MR” in “Methods” section). This is thought to occur because flipping the current leads to flipping the direction of the spin–orbit effective magnetic field, and thus, the sign of ***M*** flips. Two: There is no hysteresis. A similar shape of MR is observed between + scan and − scan. From this, we can guess that the localized spins are not ordered by the external magnetic field, but their directions are determined by spin–orbit effective magnetic field through the current. Three: In the *H*_z_ measurement, flat MR is observed when *H*_z_ is small (from − 0.3 to + 0.3 T) in the current region where linear MR is observed in the *H*_y_ measurement (Fig. 5e,j,o). This is because ***M*** is oriented in the in-plane direction, but ***H*** is out of plane; thus, ***M***·***H*** = 0. Moreover, when a large magnetic field is applied, ***M*** is released from the domination by current and is oriented to *H*_z_, and thus, the MR curve shows a kink (see MR data at 10 μA in Supplementary Fig. 2).

For the *H*_y_ measurements, linear MR starts to be observed when the positive MR starts to weaken (indicated by arrows in Fig. 5h–j,m–o). This indicates that the current-induced spin–orbit effective magnetic field changes the exchange interaction between the localized spins.

Double dips are seen for low current denoted by dips A and A′ (Fig. 3a,b). The reason for this is speculated to be that the magnetic domain is not single-domain but is multidomain, or likely because the magnetization direction is variously changed.

The disappearance of MR above 0.1 mA is probably because the localized 4*d* levels are fully occupied or because spin-dependent scattering does not occur because of the high current intensity.

Our results are related to recent progress of 2D ferromagnets, recommend 4*d* transition metal compounds as materials for spintronics, and indicate that the arrangement of polycrystalline structures unveils hidden characteristics and phenomena related to the interactions between localized spins and itinerant electrons.

## Methods

### Fabrication

We deposited a polycrystalline MoS_2_ film (7.5 nm) on a SiO_2_ (400 nm)/Si (0.7 mm) substrate using radio-frequency (RF) magnetron sputtering (EIKO ENGINEERING, LTD.) with a 4 N-purity MoS_2_ target (Matsurf Technologies Inc.). The sputtering conditions were as follows: the substrate temperature was 450 °C, Ar pressure was 0.35 Pa with 7 sccm flow, the substrate-target distance was 180 mm, and the RF power was 40 W. After deposition, we deposited an Al_2_O_3_ layer (2 nm) on the sample using atomic layer deposition (Fiji Inc.) at 300 °C with (CH_3_)_3_Al (trimethylaluminium; TMA) and H_2_O as precursors. Next, we deposited Ag pads (50 nm thickness) as electrical contacts using a current-heating vacuum evaporation tool with a shadow mask placed in front of the sample. The Ag pad pattern was a 1.8 mm × 1.8 mm square array with a 1.2 mm space. Finally, we cut the sample into small specimens, including 2 Ag pads, and bonded Au wires on them to connect to the electrodes in a sample holder.

### Magnetoresistance measurement

We performed magnetoresistance measurements using our custom-made 4 K cryostat equipment and Keithley 2400 as a source measure unit. The magnetic field was first applied at + 0.8 T and swept towards − 0.8 T at a 20 mT step (+ scan). After that, the field was swept from − 0.8 to + 0.8 T (− scan). Each magnetoresistance curve was normalized by dividing by the average value of the curve (*R* − *R*_ave_)/*R*_ave_ × 100.

### Data analysis

To extract physical values from MR curves, we used the following equation for positive MR:

1 $$\frac{R-{R}_{AVE}}{{R}_{AVE}}\left(\%\right)=\sum_{i}\frac{{c}_{i}^{2}{\left(H-{H}_{0}^{i}\right)}^{2}}{1+{d}_{i}^{2}{\left(H-{H}_{0}^{i}\right)}^{2}},$$

where *H*_0_ is the centre position of positive MR. The summation of multiple curves was performed because the curve structure was not simple, but we discussed only the parameters for dips B and C. The fitting results and each component of the curves are shown in Supplementary Figs. 2–7.

### Calculation for spin split energy

We estimated the spin-split energy of the 4*d* band from the d values by using equation^44,48^,

$${d}^{2}=\frac{{\left({\sigma }_{1}{\mu }_{2}-{\sigma }_{2}{\mu }_{1}\right)}^{2}}{{({\sigma }_{1}+{\sigma }_{2})}^{2}},$$

in which subscripts 1 and 2 represent up and down spins, respectively. In 2D system, the band energy is described by electron density,

$${E}_{i}=\frac{2\pi {\hslash }^{2}}{{m}^{*}}{n}_{i} \left(i=1, 2\right),$$

where μ, n, ћ, m^*^ correspond to the mobility, two-dimensional electron density, plank constant, and effective mass of electron, respectively. The relation between conductivity and electron density is

$${\sigma }_{i}=q{n}_{i}{\mu }_{i}, \left(i=1, 2\right).$$

Thus, d value can be modified using above equations,

2 $$d=\frac{1}{\frac{{n}_{1}}{{\mu }_{2}}+\frac{{n}_{2}}{{\mu }_{1}}}\left({n}_{1}-{n}_{2}\right)=\frac{1}{\frac{{n}_{1}}{{\mu }_{2}}+\frac{{n}_{2}}{{\mu }_{1}}}\frac{{m}^{*}}{{2\pi \hslash }^{2}}\Delta E\approx \frac{\mu }{n}\frac{{m}^{*}}{{2\pi \hslash }^{2}}\Delta E,$$

where ΔE represents spin split energy of the 4*d* band. With the assumption of μ = 0.01 cm^2^/V/s, n = 10^11^/cm^2^ for dip B, n = 2 × 10^11^/cm^2^ for dip C and m^*^ = m_0_ (electron mass), ΔE is estimated to be 2.5 meV and 100 meV for dips B (d/μ_0_ = 4 m^2^/V/s) and C (d/μ_0_ = 80 m^2^/V/s), respectively. Here, μ_0_ is the vacuum permeability.

### Calculation for linear MR

To clearly observe linear MR, we examined odd functions by using [MR_+_(H) − MR_−_(-H)]/2, where MR_+_ and MR_-_ correspond to the MR observed in the + scan and − scan measurements, respectively. The extracted odd function data are shown in Supplementary Figs. 8–10. The slope of odd function was numerically obtained by fitting the odd functions to a linear equation *y* = *ax* + *b*. The obtained values are shown in Fig. 5e,j,o.

### Calculation for the density of magnetic Mo atoms

Since there are 1 + 6/3 = 3 Mo atoms in a regular hexagon with one side length *a* = 3.16 Å, we calculated the number of Mo atoms in a square centimeter in monolayer MoS_2_:

$$\frac{3}{\frac{3\sqrt{3}}{2}{a}^{2}}=1.156 \times {10}^{15} \,{\text{cm}}^{-{2}}.$$

Similarly, the number of Mo atoms in a centimeter along out-of-plane direction was calculated:

$$\frac{1}{6.55\, {\text{{\AA}}} }=1.527 \times {10}^{7}\,{\text{cm}}^{-{1}}.$$

Thus, the density of Mo atoms in multilayer MoS_2_ was calculated:

3 $$1.156 \times {10}^{15}\,{\text{cm}}^{-{2}}\times 1.527\times {10}^{7}\,{\text{cm}}^{-{1}}=1.765\times {{10}^{22}\, \text{Mo/cm}}^{3}.$$

The saturation magnetization in Ref.^11^ was described using Bohr magneton *μ*_B_:

4 $$1\, \text{emu/}{\text{cm}}^{3} =1.078\times {10}^{20}\, {\mu }_{B}/{\text{cm}}^{3}.$$

This means the number of spin 1/2 in a cubic centimeter. The density of magnetic Mo atoms was calculated by dividing Eq. (4) with Eq. (3):

$$\frac{1.078\times {10}^{20}\, {\mu }_{B}/{\text{cm}}^{3}}{1.765\times {{10}^{22} \,{\text{Mo}}/{\text{cm}}}^{3}}=0.611\times {10}^{-2}\, {\mu }_{B}/\text{Mo.}$$

This means that 0.61% of Mo atoms have one spin 1/2.

## Supplementary Information

Supplementary Figures.

Supplementary Information.

## Data Availability

All data generated during this study are included in Supplementary Information files.
