# Supplementary material for: Ferromagnetism modulation by ultralow current in a two-dimensional polycrystalline molybdenum disulphide atomic layered structure
Source: Sci Rep. 2022 Oct 13;12:17199. doi: 10.1038/s41598-022-22113-3 (PMC9562137; doi:10.1038/s41598-022-22113-3)
Supplement: Supplementary file 1 — Supplementary Figures. [file 41598_2022_22113_MOESM1_ESM.pdf]

Supplementary Information

Ferromagnetism modulation by ultralow current in a two-dimensional polycrystalline molybdenum disulphide atomic layered structure

Iriya Muneta[1*], Takanori Shirokura[2], Pham Nam Hai[2], Kuniyuki Kakushima[1], Kazuo Tsutsui[3], and Hitoshi Wakabayashi[1]

[1] Department of Electrical and Electronic Engineering, Tokyo Institute of Technology, Yokohama, Japan

[2] Department of Electrical and Electronic Engineering, Tokyo Institute of Technology, Tokyo, Japan

[3] Laboratory for Future Interdisciplinary Research of Science and Technology, Tokyo Institute of Technology, Yokohama, Japan

Corresponding: *muneta@ee.e.titech.ac.jp

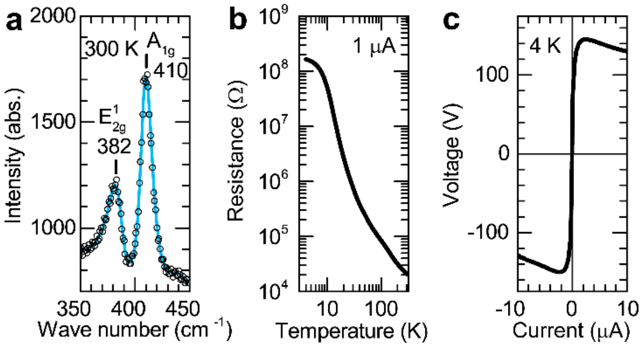

Supplementary Figure 1 | Raman and transport measurement results. a, Raman spectroscopy data measured at 300 K. Circles indicate measurement points, and the solid curve is obtained by smoothing the measurement points using the Savitzky–Golay method with the following parameters: 10 points of window and 2 polynomial order. **b**, Temperature dependence of resistance. The applied current is 1 μA. **c**, Voltage-current characteristics measured at 4 K.

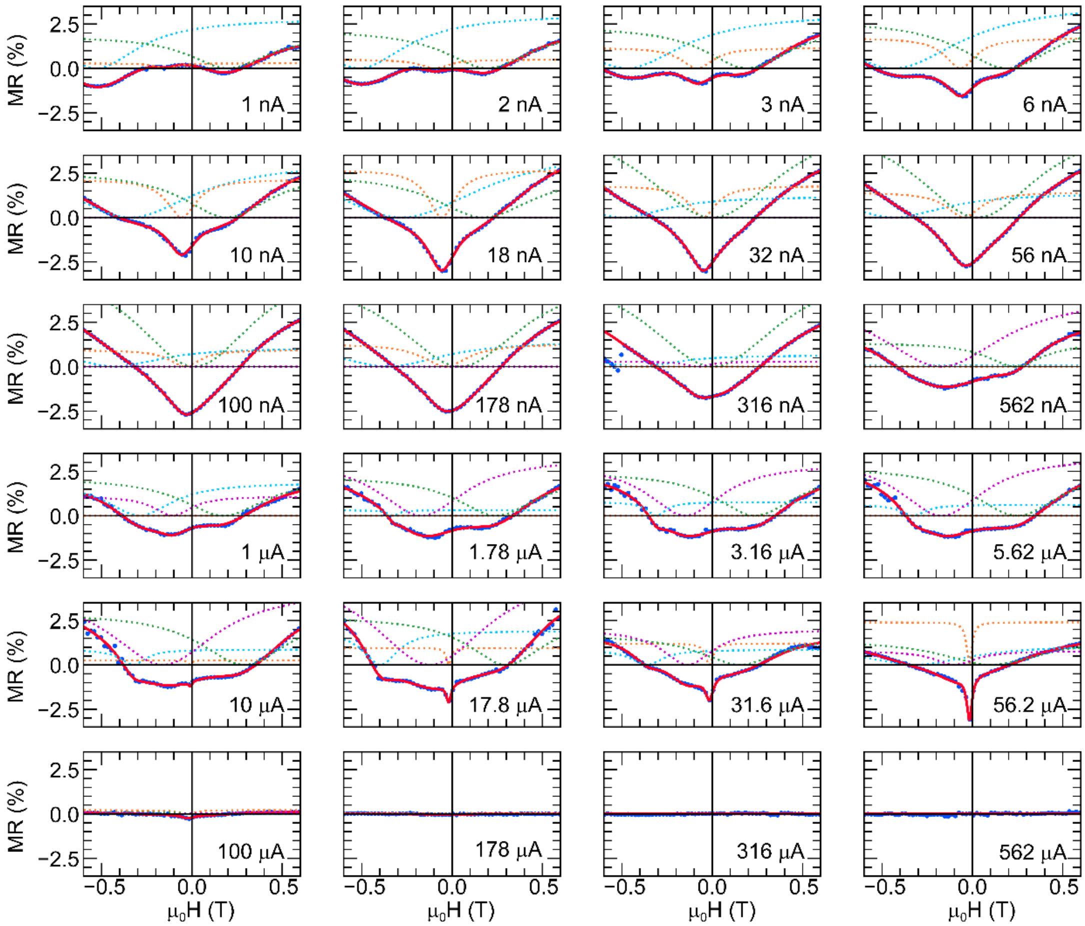

Supplementary Figure 2 | Magnetoresistance in H_z +scan measurement. Blue dots indicate measured data. Red solid curves are obtained by fitting the data to Equation 1. Light blue, orange, green and purple dotted curves indicate positive MR components corresponding to *i* in Equation 1.

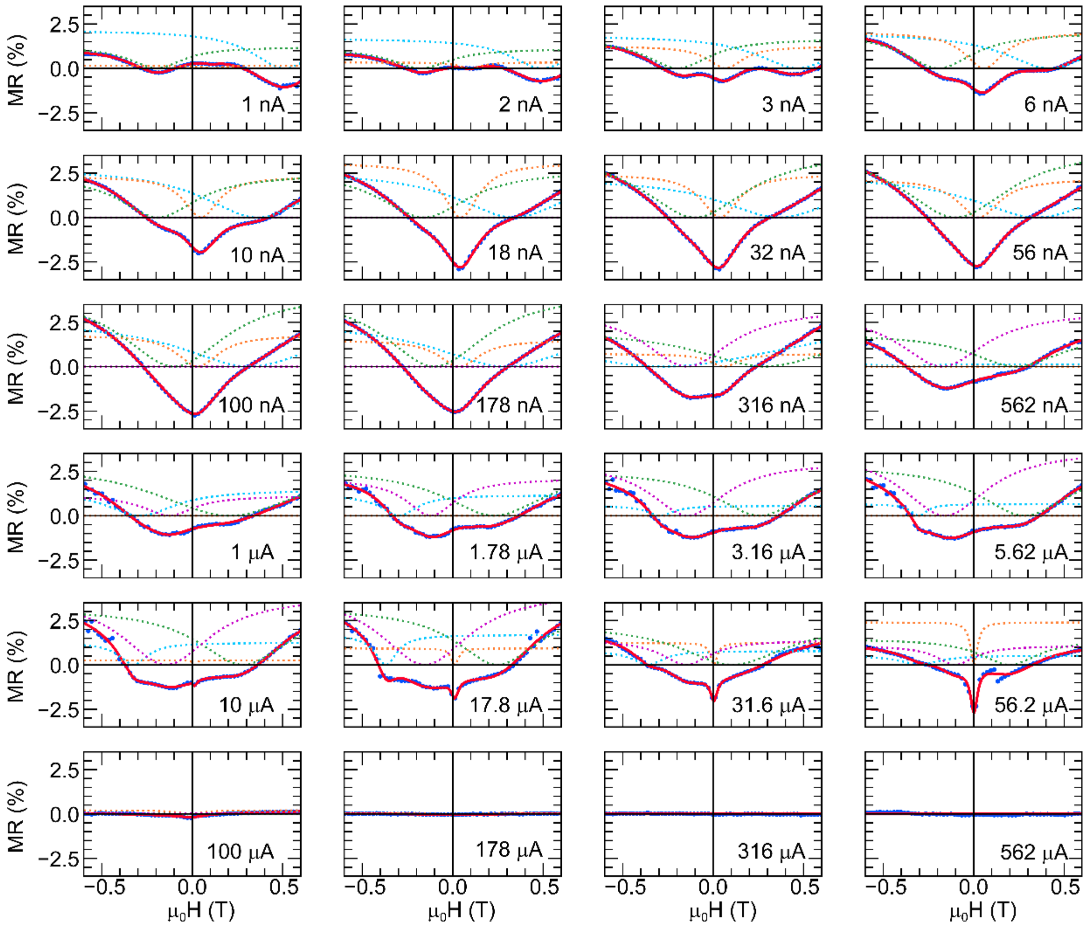

Supplementary Figure 3 | Magnetoresistance in H_z –scan measurement. Blue dots indicate measured data. Red solid curves are obtained by fitting the data to Equation 1. Light blue, orange, green and purple dotted curves indicate positive MR components corresponding to i in Equation 1.

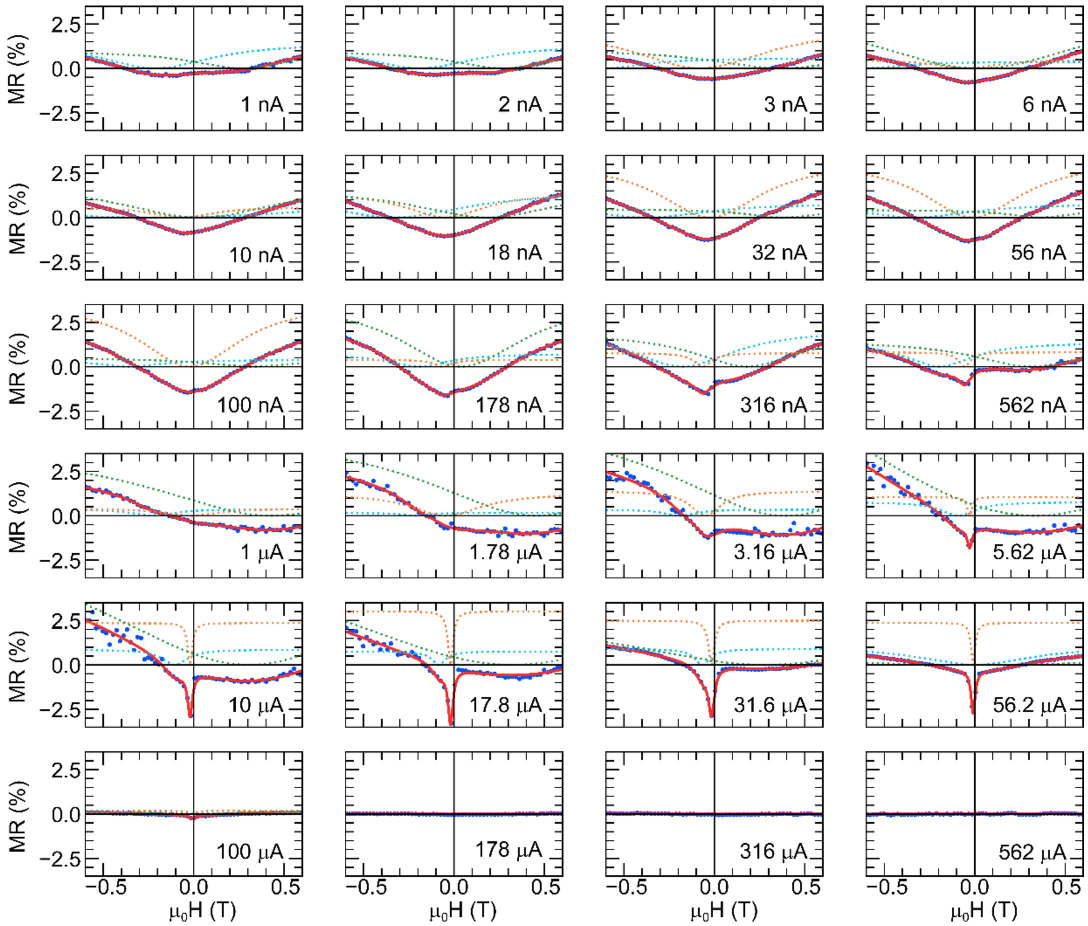

Supplementary Figure 4 | Magnetoresistance in H_y +scan $+I_x$ measurement. Blue dots indicate measured data. Red solid curves are obtained by fitting the data to Equation 1. Light blue, orange and green dotted curves indicate positive MR components corresponding to *i* in Equation 1.

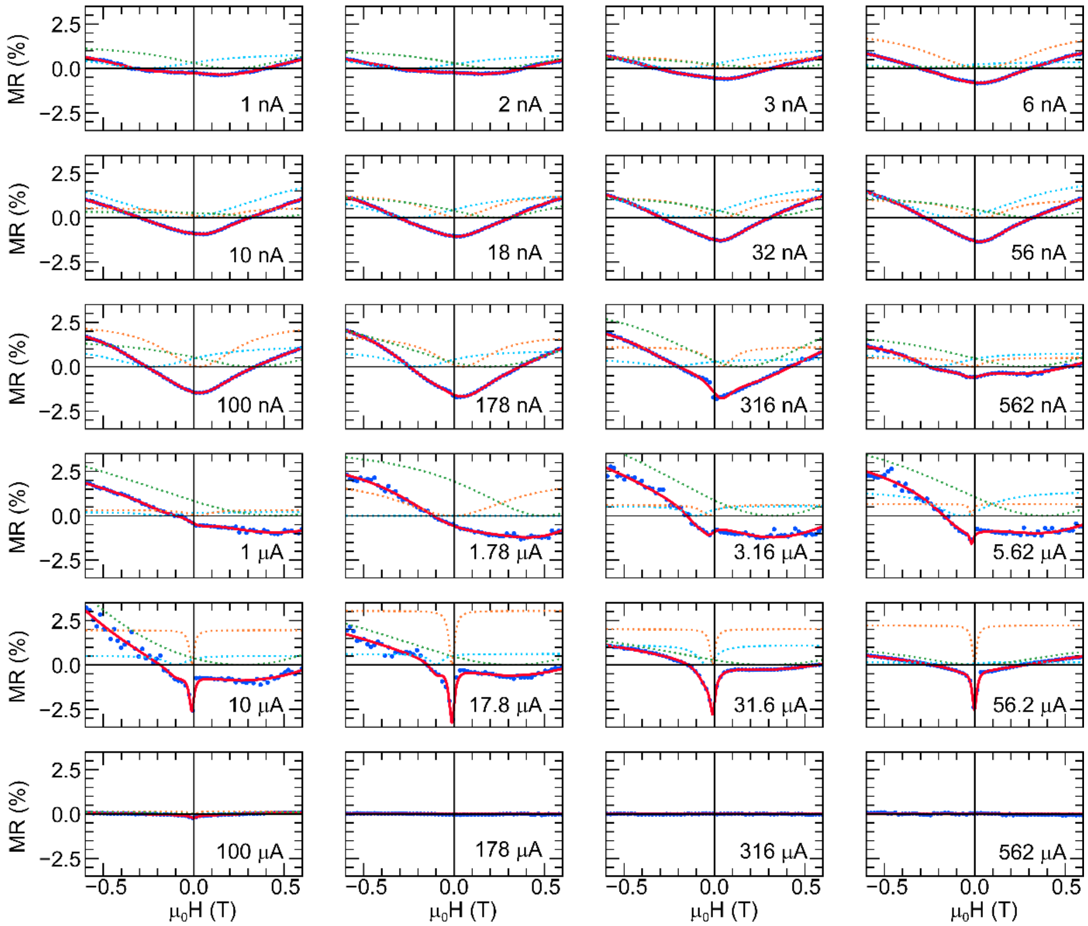

Supplementary Figure 5 | Magnetoresistance in H_y –scan $+I_x$ measurement. Blue dots indicate measured data. Red solid curves are obtained by fitting the data to Equation 1. Light blue, orange and green dotted curves indicate positive MR components corresponding to i in Equation 1.

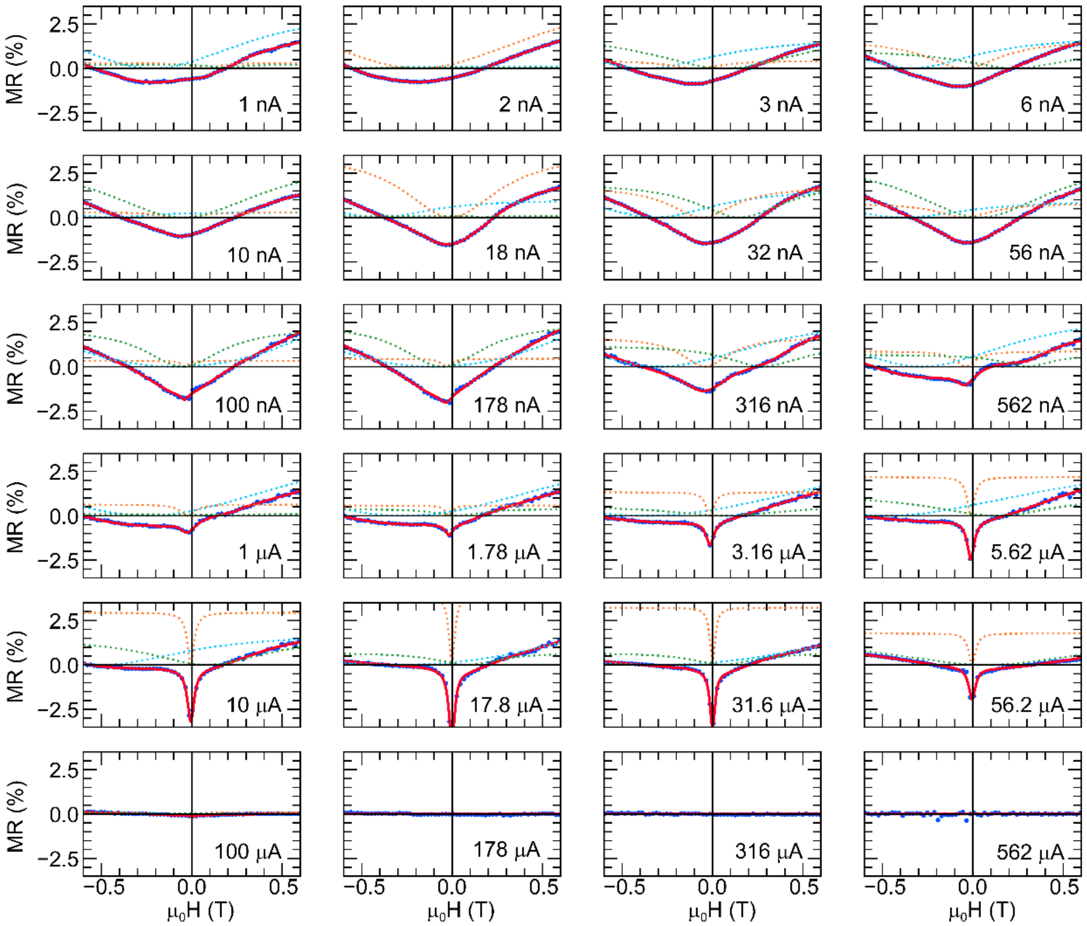

Supplementary Figure 6 | Magnetoresistance in H_y +scan $-I_x$ measurement. Blue dots indicate measured data. Red solid curves are obtained by fitting the data to Equation 1. Light blue, orange and green dotted curves indicate positive MR curves corresponding to *i* in Equation 1.

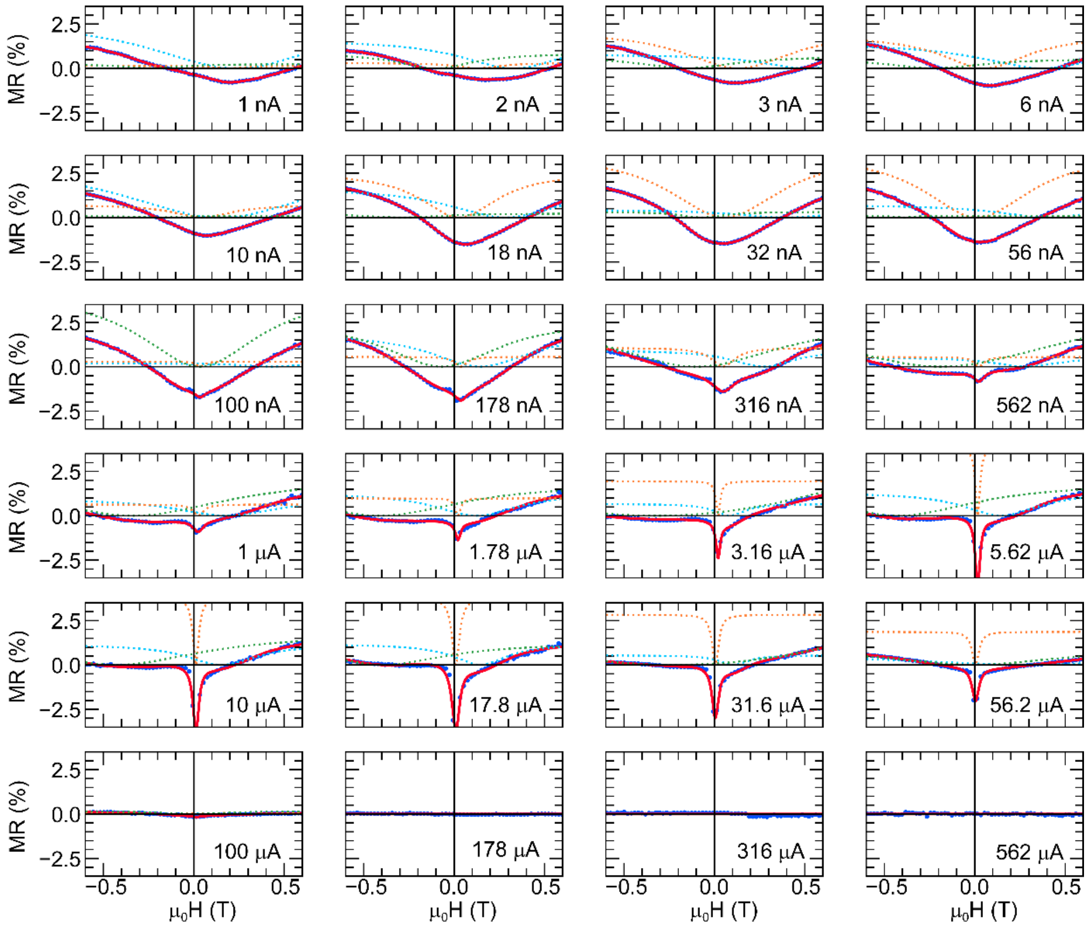

Supplementary Figure 7 | Magnetoresistance in *H_y* –scan –*I_x* measurement. Blue dots indicate measured data. Red solid curves are obtained by fitting the data to Equation 1. Light blue, orange and green dotted curves indicate positive MR components corresponding to *i* in Equation 1.

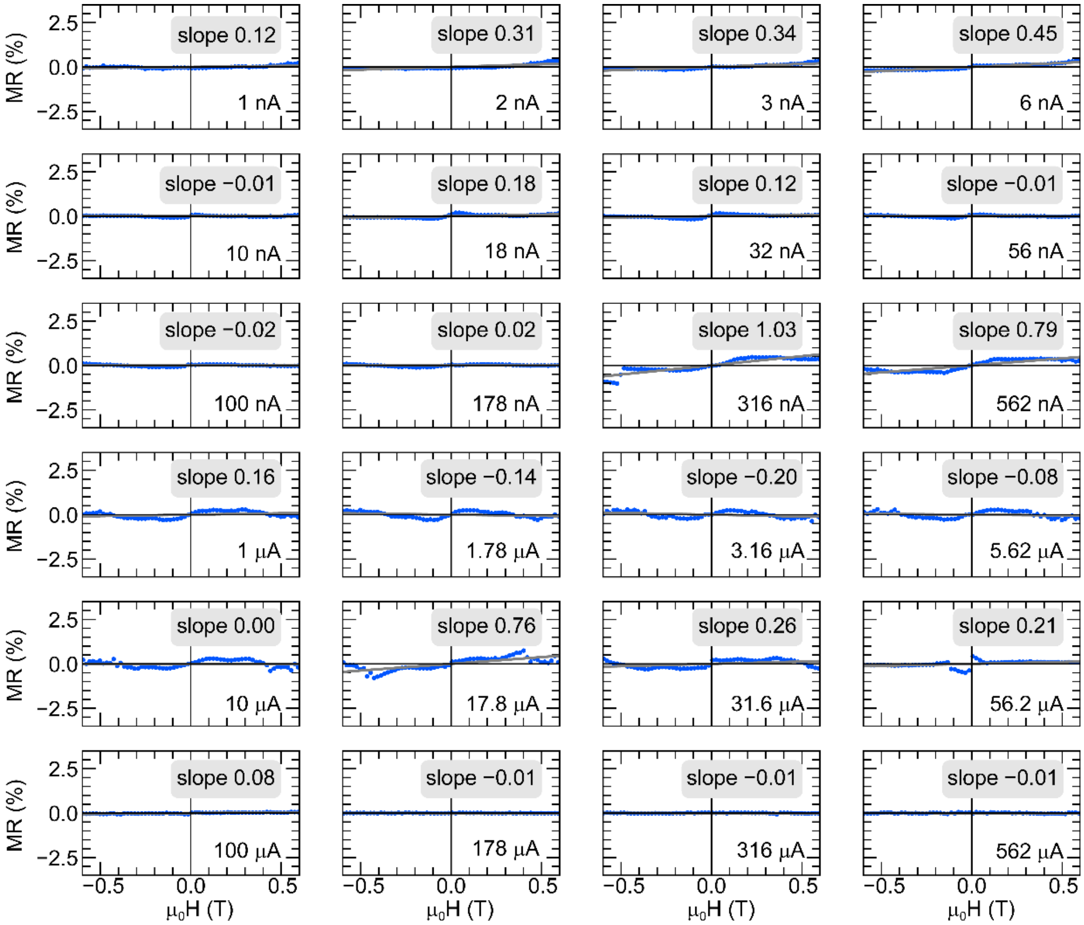

Supplementary Figure 8 | Odd function obtained from magnetoresistance data in H_z measurement.

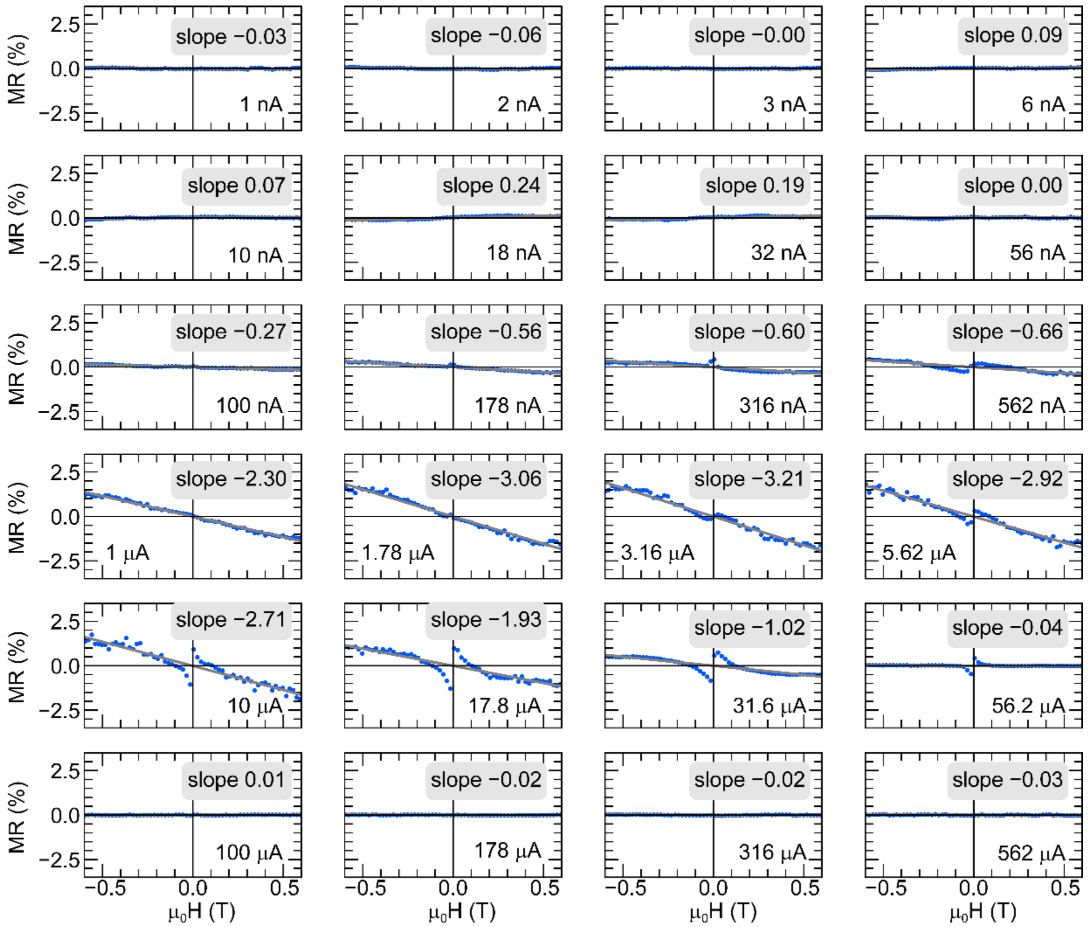

Supplementary Figure 9 | Odd function obtained from magnetoresistance data in the H_y +I_x measurement.

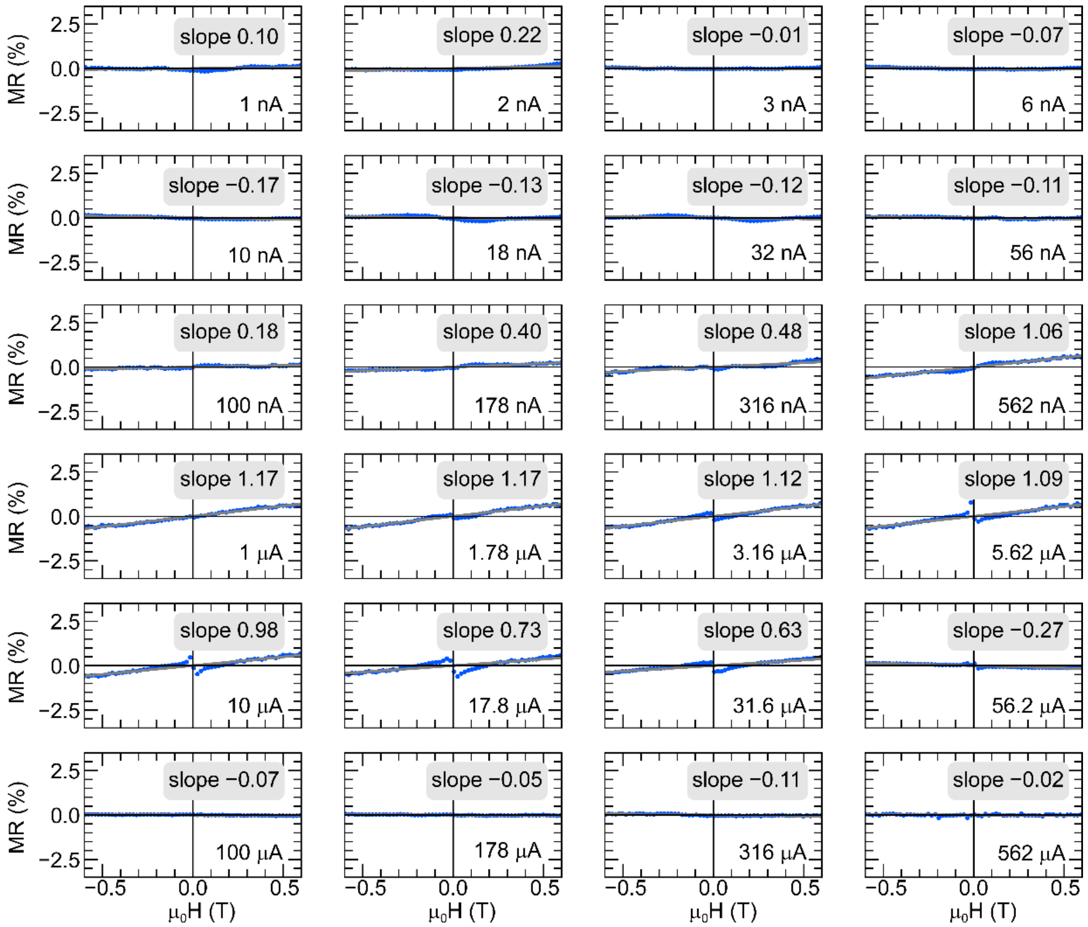

Supplementary Figure 10 | Odd function obtained from magnetoresistance data in the H_y -I_x measurement.
